# The impact of COVID-19 on open access publishing in radiology and nuclear medicine: an in-depth analysis

**DOI:** 10.25122/jml-2023-0075

**Published:** 2023-07

**Authors:** Lna Malkawi, Reem Hassan, Mohammad Ali Alshrouf, Nosaiba Al-Ryalat, Saif Aldeen AlRyalat

**Affiliations:** 1 Department of Radiology, University of Jordan, Amman, Jordan; 2 Family Medicine, Primary Health Care Corporation, Doha, Qatar; 3 School of Medicine, University of Jordan, Amman, Jordan; 4 Department of Special Surgery, University of Jordan, Amman, Jordan

**Keywords:** open access publishing, publishing, COVID-19, radiology, nuclear medicine, policymakers

## Abstract

In response to the COVID-19 pandemic, numerous initiatives have been implemented to ensure open access availability of COVID-19-related articles to make published articles accessible for anyone. This study aimed to assess the impact of the COVID-19 pandemic on open-access publishing in radiology and nuclear medicine. We conducted a comprehensive analysis of articles and reviews published in these fields during the COVID-19 publishing era using the Web of Science database. We analyzed several indicators between COVID-19 and non-COVID-19 related articles, including the number and percentage of open-access articles, the top ten cited articles, and the number of reviews. In total, 67,100 articles were published in radiology and nuclear medicine between January 2020 and June 2022. Among those, more than half (51.1%) were open-access articles. Among these publications, 2,336 were COVID-19-related, and 64,764 were non-COVID-19-related. However, articles related to COVID-19 had an open access rate of 91.5%, compared to only 49.6% of the non-COVID-19-related articles. Moreover, COVID-19-related articles had a higher percentage of highly cited and hot papers compared to articles not related to COVID-19. Moreover, most highly cited studies were related to chest computerized tomography (CT) scan findings in COVID-19 patients. The findings emphasize the significant proportion of open access COVID-19-related publications in radiology and nuclear medicine, facilitating widespread and timely access to everyone.

## INTRODUCTION

In late 2019, the emergence of a novel coronavirus strain known as SARS-CoV-2 led to the rapid spread of a respiratory infectious disease called COVID-19, resulting in a global pandemic [1]. Following that, Chinese scholars published a series of descriptive studies on the clinical features of COVID-19, which were quickly followed by articles published worldwide, with the first article describing the clinical and radiological characteristics and management of the disease published in China [2]. The pandemic prompted a significant mobilization of financial resources by governments and private organizations worldwide, leading to a shift in research focus toward COVID-19-related studies and the allocation of funding for related projects [3, 4].

The emergence of COVID-19 changed the research landscape, with the disease becoming a central topic across various fields [5]. In radiology and nuclear medicine, COVID-19 quickly became one of the most extensively studied subjects in 2020, reflected by the high citation rates of COVID-19-related articles [6, 7]. Several journals made changes, considering it was critical to get information about COVID-19 out quickly. This led many journals, including Radiology, European Radiology, American Journal of Roentgenology, and Journal of the American College of Radiology, to build a dedicated resource center, which led to their COVID-19 articles getting more attention and citations [8, 9].

Open-access publishing was essential for researchers and people all over the world to deal with the COVID-19 pandemic in a timely manner. In January 2020, the Wellcome Trust issued a call that publishers should make journal articles related to COVID-19 open access or free to read immediately upon publication [10]. Several authorities worldwide agreed to the open access mandate for COVID-19-related publications [11]. These mandates positively impacted knowledge dissemination among researchers and physicians, especially in institutions with limited access to non-open-access articles. In this study, we assessed the impact of the COVID-19 era on open-access publishing in radiology and nuclear medicine. Such trend analysis can inform researchers and policymakers on ways to improve the current research landscape in this field [12].

## MATERIAL AND METHODS

### Overview

This study employed a bibliometric analysis of literature in radiology and nuclear medicine. We examined articles and reviews published during the COVID-19 publishing era in this field. We conducted our search using the Web of Science database because of its field categorization features, advanced open-access discovery features, and high-quality article indexing strategy [13]. We reported the results according to the PRISMA statement and its extensions [14].

Within the Web of Science database, open access articles are classified into three categories: gold open access, referring to articles published in journals indexed in the Directory of Open Access Journals (DOAJ); green open access, indicating articles that have an embargo period following their initial publication and are exclusively accessible to subscribers during this period; and bronze, denoting articles that are freely available to read on the publisher's website but are not published under an open access license. It has also developed advanced tools to discover open-access articles with peer-reviewed versions legally hosted in open repositories. The open access status is updated weekly in the database [15].

### Search strategy

The search strategy was conducted on June 6^th^, 2022, and encompassed articles and reviews indexed in the Web of Science database under the category “radiology, nuclear medicine, and medical imaging.” The search included articles indexed in all Web of Science indices, including Science Citation Index Expanded (SCI-EXPANDED), Emerging Sources Citation Index (ESCI), and Social Sciences Citation Index (SSCI). We restricted the search to English-language articles and focused on “article” or “review” article types within the Web of Science category “radiology, nuclear medicine, and medical imaging.”

To specifically search for COVID-19 articles, the following search query was used: Title (TI) = (Coronavirus OR COVID19 OR COVID-19 OR nCOVID19 OR SARS-CoV-2 OR “SARS COV 2” OR Orthocoronavirinae) OR Author Keywords (AK) = (Coronavirus OR COVID19 OR COVID-19 OR nCOVID19 OR SARS-CoV-2 OR “SARS COV 2” OR Orthocoronavirinae).

In total, 2,343 articles resulted from the search, of which 2,336 were published between 2020 and 2022 (up to June). More than 99.7% of search results were between 2020 and 2022 (up to June 6^th^). Subsequently, we restricted the search to articles between January 2020 and June 2022.

To search for non-COVID-19-related articles in the specified period, we combined both searches above using the “NOT” operator to exclude COVID-19-related publications from all radiology and nuclear medicine publications between January 2020 and June 2022. We searched for articles published within that period and retrieved 64,764 non-COVID-19-related articles.

### Variables

We analyzed several indicators between COVID-19 and non-COVID-19 related articles, including the number and percentage of open-access articles, the top ten cited articles, and the number of reviews. The articles were arranged in descending order based on the number of citations. We compared the year of publication, journal of publication, publisher, institution, and country of origin between groups. In addition, we obtained the following indices from the Web of Science database: (1) Highly Cited Papers (HCPs), which are papers that perform in the top 1% based on the number of citations received when compared to other papers published in the same field in the same year, and (2) Hot Papers, which are papers published in the last two years that are receiving citations quickly after publication. These papers have been cited enough times in the most recent bimonthly period to place them in the top 0.1% compared to papers in the same field and added to the database in the same period. We used statistical and visualization tools provided by the Web of Science databases to draw the results of this study.

## RESULT

67,100 articles were published in radiology and nuclear medicine between January 2020 and June 2022. Over half of the articles (34,922 (51.1%)) were open access. Of the total publications, 2,336 were COVID-19-related, and 64,764 were non-COVID-19-related. Table 1 compares COVID-19-related and non-COVID-19-related publications regarding count, open access, highly cited, hot papers, and review articles.

**Table 1:** Comparison of COVID-19 and non-COVID-19 related publications in terms of count, open access, highly cited and hot papers, and review articles

Variable	COVID-19 related articles	Non-COVID-19 related articles
Count	2,336 (3.5)	64,764 (96.5)
Open access count	2,134 (91.4)	32,132 (49.6)
Highly cited papers	192 (8.2)	185 (0.3)
Hot papers count	11 (0.5)	6 (0.001)
Review articles count	342 (14.6)	6,123 (9.5)

Data are represented in n (%).

### COVID-19-related articles

Out of the 2,336 COVID-19-related articles analyzed, nearly half (48%) were published in 2021. Harvard University emerged as the most common affiliation, contributing to 229 (9.8%) publications, while the United States stood out as the most frequently published country with 788 (33.7%) articles. Clinical Imaging was the leading journal in terms of publication count, with 116 (5%) articles, and Springer Nature emerged as the top publisher, with 698 (29.9%) articles. Detailed characteristics of COVID-19-related articles in radiology and nuclear medicine can be found in Table 2.

**Table 2:** Characteristics of COVID-19-related articles in the field of radiology and nuclear medicine

Variable	Categories	Number (%)
Year	2022	266 (11.4)
	2021	1,121 (48.0)
	2020	949 (40.6)
Affiliation	Harvard University	229 (9.8)
	The League of European Research Universities	195 (8.3)
	Massachusetts General Hospital	85 (3.6)
	The Egyptian Knowledge Bank	82 (3.5)
	Udice French Research Universities	71 (3.0)
Journal	Clinical Imaging	116 (5)
	European Radiology	114 (4.9)
	Egyptian Journal of Radiology and Nuclear Medicine	106 (4.5)
	Radiology	82 (3.5)
	Academic Radiology	73 (3.1)
Publisher	Springer Nature	698 (29.9)
	Elsevier	648 (27.7)
	Wiley	147 (6.3)
	SAGE	69 (3.0)
	Radiological Society of North America	66 (2.8)
Country	USA	788 (33.7)
	China	346 (14.8)
	Italy	333 (14.3)
	UK	197 (8.4)
	India	131 (5.6)

Among the 2,336 articles published, 2,137 (91.5%) were open-access articles. For the remaining 199 non-open access articles identified in the Web of Science database, we conducted a manual search using Google Scholar to assess their accessibility. Among these 199 non-open access articles extracted from the Web of Science, only 65 (32.7%) were not accessible through the manual search. Overall, 15 articles were published by Wolters Kluwer Health, 12 by Bentham Science Publishers, and 8 by IOP Science. None of the non-open access articles were published by Springer Nature, the top publisher of COVID-19-related articles.

The most highly cited article was related to chest computerized tomography (CT) scan findings in COVID-19 patients, titled “Correlation of Chest CT and RT-PCR Testing for Coronavirus Disease 2019 (COVID-19) in China: A Report of 1014 Cases", which received 2,831 citations [16]. Table 3 shows the top 10 cited papers on COVID-19 in radiology and nuclear medicine [16-25].

**Table 3:** Top 10 cited papers related to COVID-19 in radiology and nuclear medicine

Rank	Authors	Article title	Number of citations	Journal
1	Ai *et al*. (2020) [16]	Correlation of Chest CT and RT-PCR Testing for Coronavirus Disease 2019 (COVID-19) in China: A Report of 1014 Cases	2,831	Radiology
2	Fang *et al*. (2020)[17]	Sensitivity of Chest CT for COVID-19: Comparison to RT-PCR	1,526	Radiology
3	Pan *et al*. (2020) [18]	Time Course of Lung Changes a Chest CT during Recovery from Coronavirus Disease 2019 (COVID-19 )	1,163	Radiology
4	Xie *et al*. (2020) [19]	Chest CT for Typical Coronavirus Disease 2019 (COVID-19) Pneumonia: Relationship to Negative RT-PCR Testing	1,108	Radiology
5	Zu *et al*. (2020) [20]	Coronavirus Disease 2019 (COVID-19) A Perspective from China	800	Radiology
6	Song *et al*. (2020) [21]	Emerging 2019 Novel Coronavirus (2019-nCoV) Pneumonia	698	Radiology
7	Apostolopoulos & Mpesiana (2020) [22]	Covid-19: automatic detection from X-ray images utilizing transfer learning with convolutional neural networks	650	Physical and Engineering Sciences in Medicine
8	Zhao *et al*. (2020) [23]	Relation Between Chest CT Findings and Clinical Conditions of Coronavirus Disease (COVID-19) Pneumonia: A Multicenter Study	613	American Journal of Roentgenology
9	Ye *et al*. (2020) [24]	Chest CT manifestations of new coronavirus disease 2019 (COVID-19): a pictorial review	611	European Radiology
10	Bai *et al*. (2020) [25]	Performance of Radiologists in Differentiating COVID-19 from Non-COVID-19 Viral Pneumonia at Chest CT	610	Radiology

### Non-COVID-19-related articles

Of the 64,764 non-COVID-19-related articles, 44% were published in 2021. The League of European Research Universities was the most common affiliation with 9,029 (13.9%) publications, with the USA being the most commonly published country with 21,101 (32.6%). Neuroimage was the journal that published the most, with 2,360 (3.6%) articles, and Elsevier emerged as the top publisher, with 17,114 (26.4%) articles. Table 4 details the characteristics of non-COVID-19 articles in radiology and nuclear medicine.

**Table 4:** Column I. Characteristics of non-COVID-19 articles in radiology and nuclear medicine

Variable	Categories	Number (%)
Year	2022	9,029 (13.9)
	2021	28,499 (44)
	2020	949 (1.5)
Affiliation	The League of European Research Universities	6,523 (13.9)
	Harvard University	4,012 (6.2)
	University of California	2,164 (3.3)
	University of London	1,791 (2.8)
	University of Texas	1,787 (2.8)
Journal	Neuroimage	2,360 (3.6)
	European Radiology	2,165 (3.3)
	Medical Physics	1,556 (2.4)
	Physics in Medicine and Biology	1,252 (1.9)

**Table 4:** Column II. Characteristics of non-COVID-19 articles in radiology and nuclear medicine

Variable	Categories	Number (%)
	Abdominal Radiology	1,146 (1.8)
Publisher	Elsevier	17,114 (26.4)
	Springer Nature	15,902 (24.6)
	Wiley	7,821 (12.1)
	IOP Publishing	2,020 (3.1)
	SAGE	1,897 (2.9)
Country	USA	21,101 (32.6)
	China	10,900 (16.8)
	Germany	6,046 (9.3)
	UK	4,793 (7.4)
	Japan	4,163 (6.4)

The article with the most citations, 1172, was about accelerated imaging techniques using Wave-CAIPI susceptibility-weighted imaging, titled “Wave-CAIPI susceptibility-weighted imaging achieves diagnostic performance comparable to conventional susceptibility-weighted imaging in half the scan time” [26]. Table 5 shows the top 10 cited papers not related to COVID-19 in radiology and nuclear medicine [26-35].

**Table 5:** Top 10 cited papers not related to COVID-19 in radiology and nuclear medicine

Rank	Authors	Article title	Number of citations	Journal
1	Chung *et al*. (2020) [26]	Wave-CAIPI susceptibility-weighted imaging achieves diagnostic performance comparable to conventional susceptibility-weighted imaging in half the scan time	1,172	European Radiology
2	Zwanenburg *et al*. (2020) [27]	The Image Biomarker Standardization Initiative: Standardized Quantitative Radiomics for High-Throughput Image-based Phenotyping	632	Radiology
3	ICNIRP (2020) [28]	Guidelines for Limiting Exposure to Electromagnetic Fields (100 kHz to 300 GHz)	346	Radiology
4	Zhou *et al*. (2021) [29]	UNet++: Redesigning Skip Connections to Exploit Multiscale Features in Image Segmentation	234	IEEE Transactions on Medical Imaging
5	Kramer (2020) [30]	Standardized cardiovascular magnetic resonance imaging (CMR) protocols: 2020 update	207	Journal of Cardiovascular Magnetic Resonance
6	Rolls *et al*. (2020) [31]	Automated anatomical labelling atlas 3	191	NeuroImage
7	Chen *et al*. (2020) [32]	Comparison of [^68^Ga]Ga-DOTA-FAPI-04 and [^18^F] FDG PET/CT for the diagnosis of primary and metastatic lesions in patients with various types of cancer	177	European Journal of Nuclear Medicine and Molecular Imaging
8	Schulz-Menger *et al*. (2020) [33]	Standardized image interpretation and post-processing in cardiovascular magnetic resonance-2020 update Society for Cardiovascular Magnetic Resonance (SCMR): Board of Trustees Task Force on Standardized Post-Processing	174	Journal of Cardiovascular Magnetic Resonance
9	Tajbakhsh *et al*. (2020) [34]	Embracing imperfect datasets: A review of deep learning solutions for medical image segmentation	148	Medical Image Analysis
10	Mayerhoefer *et al*. (2020) [35]	Introduction to Radiomics	133	Journal of Nuclear Medicine

## DISCUSSION

Mandates for open-access publishing during the past few years resulted in a high percentage of open-access articles related to COVID-19 in radiology and nuclear medicine. While radiology and nuclear medicine articles generally had an open access rate of 51.1%, articles related to COVID-19 had an open access rate of 91.5%. Most publishers complied with open access deposition of COVID-19 articles, and only a few publishers had non-open access COVID-19-related articles. Moreover, we observed that COVID-19-related articles had a higher percentage of highly cited and hot papers than articles unrelated to COVID-19. This finding aligns with a previous study showing that interrelated topics tend to co-cite each other in radiology and imaging [36]. Interestingly, nine of the ten most-cited COVID-19-related articles were published in China, even though the United States published twice as many articles on the subject. The significance of these findings lies in their ability to highlight the ongoing trend of open-access publishing during this critical period. This knowledge will help us plan toward greater emphasis on open-access publishing, mainly when the need for widespread knowledge sharing is crucial.

The radiology and nuclear medicine fields gradually shifted toward open-access publishing, as evidenced by the steady increase in open-access journals and their growing impact on radiology literature before the COVID-19 era [37]. The COVID-19 conference and the associated open-access movement provided the impetus for open-access publishing. We observed a high percentage of open-access COVID-19-related articles in radiology and nuclear science, with non-open-access articles being an exception in this field and for a few publishers. A recent study that compared the percentage of open-access publications in general between the COVID-19 era and the pre-COVID-19 era found that open-access publishing almost doubled during the COVID-19 era. However, open-access articles comprised only around half of the total publications [38]. Previous studies also pointed to a change in the top countries publishing research during the COVID-19 era [39], consistent with our findings that the top countries publishing COVID-19 research differ from those publishing non-COVID-19 research. Another positive impact of COVID-19 on the publishing landscape has been the notable increase in international research collaboration during this period [40].

While most COVID-19 and non-COVID-19 related publications were from the USA, nine out of the top ten cited COVID-19 articles originated from China, in contrast to only one out of the top ten cited non-COVID-19 articles. This discrepancy can be attributed to the fact that the COVID-19 outbreak initially occurred in Wuhan, China, in December 2019 [2]. A comprehensive analysis of the COVID-19 literature in 2020 revealed that research articles predominantly focused on areas such as public health response, clinical care practices, clinical characteristics, risk factors, and epidemic models focusing on the spread of the virus [5]. We found that seven of the ten most-cited COVID-19-related articles were related to CT scan findings in infected patients, whereas none of the ten most-cited non-COVID-19 articles discussed this topic. Similar results were obtained in a previous study, indicating that CT was the most frequently discussed topic in radiology journals in 2020 and strongly correlated with COVID-19 [6].

Many concerns were raised concerning the quality of the COVID-19 research papers due to the large number of retractions and withdrawals, which raised questions related to the quality of many published articles and the publication process [41-44]. It also showed that journals must balance rigor and speed to publish high-quality papers [41]. COVID-19-related publications were rapidly generated, had generally faster acceptance compared to non-COVID-19 publications, and were freely deposited as open access [39]. In a recent opinion paper, the author argued that such open-access publishing during COVID-19 paved the way for predatory open-access journals to reach more authors and disseminate more rapidly [45]. Such predatory or fake journals may attract novice authors, who may not be able to differentiate a fake journal from a legitimate one, especially with the rapid pace of acceptance and publication becoming the norm [46]. In addition, there was a shift in views and acceptance of preprints, with COVID-19 preprints being more extensively shared and cited than non-COVID-19 preprints, accounting for around 25% of COVID-19-related research in 2020 [47].

One of the main limitations of this study is its reliance on the Web of Science database as the primary data source. The aim of the bibliometric analysis is to show the trend in publications [12]. This includes searching as many databases as possible and restricting the search results through a well-designed search strategy. In this study, we included all Web of Science databases and restricted the search through the described strategy. In addition, previous studies showed that using multiple databases would complement bibliometric analysis [13]. However, other databases generally do not categorize articles by field, which would limit the use of multiple databases in the current study. Even though most high-quality articles are indexed in the Web of Science, other relevant published articles might not be indexed in the Web of Science and were not included in our study. Furthermore, when comparing older publications to newer ones, there is often a bias in favor of the older ones. Moreover, as previously recommended by a bibliometric analysis study on radiology, nuclear medicine, and medical imaging, our study included articles and reviews as distinct papers [48]. Finally, the authors would like to acknowledge that the argument to move to open access requires that these costs be addressed, especially considering there are still costs for the journal office, editors, copy editors, printing, and mailing for those journals that publish hard copy journals.

## CONCLUSION

Open-access publishing has emerged as a valuable tool for researchers and individuals worldwide, enabling them to respond rapidly to the challenges posed by the COVID-19 pandemic. Our analysis of the recently published literature demonstrated preliminary evidence of the impact of the COVID-19 era on open-access publishing in radiology and nuclear medicine. A high proportion of COVID-19-related open-access articles were published in the previous few years, facilitating their reach to everyone in a timely manner. The radiology and nuclear medicine articles related to COVID-19 had almost double the rate of open-access publications, and only a few articles were not freely available. In addition, open-access publishing played a crucial role as an authentic and trustworthy source of scientific information during the COVID-19 pandemic for researchers, policymakers, public health professionals, and the general public.
